# Comparison of a Collective Intelligence Tailored Messaging System on Smoking Cessation Between African American and White People Who Smoke: Quasi-Experimental Design

**DOI:** 10.2196/18064

**Published:** 2020-04-27

**Authors:** Jamie M Faro, Catherine S Nagawa, Jeroan A Allison, Stephenie C Lemon, Kathleen M Mazor, Thomas K Houston, Rajani S Sadasivam

**Affiliations:** 1 Department of Population and Quantitative Health Sciences University of Massachusetts Medical School Worcester, MA United States; 2 School of Medicine University of Massachusetts Medical School Worcester, MA United States; 3 Meyers Primary Care Institute University of Massachusetts Medical School Worcester, MA United States; 4 Section on General Internal Medicine Wake Forest School of Medicine Winston-Salem, NC United States

**Keywords:** machine learning, computer-tailored health communication, smoking cessation, health disparities

## Abstract

**Background:**

The Patient Experience Recommender System for Persuasive Communication Tailoring (PERSPeCT) is a machine learning recommender system with a database of messages to motivate smoking cessation. PERSPeCT uses the collective intelligence of users (ie, preferences and feedback) and demographic and smoking profiles to select motivating messages. PERSPeCT may be more beneficial for tailoring content to minority groups influenced by complex, personally relevant factors.

**Objective:**

The objective of this study was to describe and evaluate the use of PERSPeCT in African American people who smoke compared with white people who smoke.

**Methods:**

Using a quasi-experimental design, we compared African American people who smoke with a historical cohort of white people who smoke, who both received up to 30 emailed tailored messages over 65 days. People who smoke rated the daily message in terms of perceived influence on quitting smoking for 30 days. Our primary analysis compared daily message ratings between the two groups using a *t* test. We used a logistic model to compare 30-day cessation between the two groups and adjusted for covariates.

**Results:**

The study included 119 people who smoke (African Americans, 55/119; whites, 64/119). At baseline, African American people who smoke were significantly more likely to report allowing smoking in the home (*P*=.002); all other characteristics were not significantly different between groups. Daily mean ratings were higher for African American than white people who smoke on 26 of the 30 days (*P*<.001). Odds of quitting as measured by 30-day cessation were significantly higher for African Americans (odds ratio 2.3, 95% CI 1.04-5.53; *P*=.03) and did not change after adjusting for allowing smoking at home.

**Conclusions:**

Our study highlighted the potential of using a recommender system to personalize for African American people who smoke.

**Trial Registration:**

ClinicalTrials.gov NCT02200432; https://clinicaltrials.gov/ct2/show/NCT02200432

**International Registered Report Identifier (IRRID):**

RR2-10.2196/jmir.6465

## Introduction

Computer-tailored health communication (CTHC) increases the personal relevance of health messaging by matching the messages to an individual or group’s characteristics [1] and can be effective in motivating behavior change [2-8]. CTHC is traditionally accomplished using rule-based approaches in which selected variables from patients’ baseline profiles are matched to if-then tailoring rules to select messages for specific subsets of patients [1,9]. Instead of using rule-based approaches, companies like Amazon use a special class of machine learning systems (recommender systems) to select messages. These systems combine the collective intelligence of their users (ie, the observed and inferred preferences of users as they interact with the system) and their user profiles [10-12]. Our prior work [13] developed and tested a recommender system (Patient Experience Recommender System for Persuasive Communication Tailoring [PERSPeCT]) for smoking cessation. Essentially, the system selects messages to send that are more likely to motivate you because “smokers like you were influenced by messages like this one.” As reported in our prior randomized trial [13], the PERSPeCT system outperformed the standard rule-based system in terms of daily message ratings and 30-day cessation.

Recommender systems have several advantages over rule-based approaches in health communication interventions, including the ability to continuously learn from user feedback (eg, liked product, products purchased) and enhance personal relevance [9]. Consequently, an anticipated benefit is that the recommender system can be even more beneficial for tailoring content to members of minority groups who are influenced by complex factors [9], especially as developers often have difficulty selecting variables for tailoring content for these groups. In this study, we focused on testing the PERSPeCT system with African American people who smoke. African Americans are more likely to die from smoking-related diseases than whites [14]. They are also less likely to be successful at quitting because they are less likely to seek cessation support [15-18]. African American people who smoke report more risk factors for cessation difficulties including greater nicotine dependence, more depressive symptoms, and lower readiness to quit compared with other race/ethnic groups [19]. They are less likely to receive tobacco counseling from health care providers [7] or use nicotine replacement therapy (NRT) compared with white people who smoke [20]. Very few tailoring interventions have successfully targeted African American people who smoke [21]. Thus, there is a real need for interventions that motivate cessation and are personally relevant to African American people who smoke.

This study is a pilot evaluation of our original evaluation of our recommender system in a new population, African American people who smoke. In our original experiment [NCT02200432], we recruited a general population of people who smoke, and most participants (92%) who enrolled were white. In this study, we recruited only African American people who smoke (n=55), and then compared the results with white people who smoke. Understanding the differential response to PERSPeCT between white and African American people who smoke may lead to improving PERSPeCT for African American people who smoke [15]. We compared daily message ratings, intervention engagement, perceived intervention impact, and 30-day cessation.

## Methods

### Study Design

We recruited African American people who smoke (n=55) to the PERSPeCT intervention. Then, we conducted a quasi-experimental comparison of the effectiveness of PERSPeCT in the African American intervention with a nonconcurrent comparison group of white people who smoke (the historical cohort of 64 white people who smoke who had received PERSPeCT messages in a prior trial) [13]. The data collection procedure of this study mirrored the original trial. The African American person who smokes intervention was conducted between April 2017 and November 2017; whereas the historical control data were collected between October 2014 and January 2015. This study was approved by the University of Massachusetts medical school institutional review board.

### Intervention: Patient Experience Recommender System for Persuasive Communication Tailoring

The description of the recommender system is detailed elsewhere [13,22,23]. Briefly, the system includes a messages database and machine learning algorithm. The message database includes 261 expert or peer written messages [24]. Expert-written messages were composed through an iterative group review process guided by theoretical frameworks and existing smoking cessation guidelines [25]. Peer-written messages were composed by current and former people who smoke responding to an online survey. Messages included motivational content such as reasons to quit and tips and strategies to support a quit attempt, such as substitution and distraction and use of NRT.

The system sent one message daily for 30 days. Messages were delivered to the person who smokes’s email address. At enrollment, we explained to people who smoke how the system worked. Each day, the participant was asked to rate the message by replying with a rating from 1 (strongly disagree) to 5 (strongly agree). Note that ratings are not required for the system to send more messages, however, the more ratings the system receives, the more personalized the daily messages can become. The daily messaging system was supported by a website with additional information. The website included functions designed to support cessation induction and maintenance such as information on smoking risks, tips on communicating with family members, and a library of cessation materials.

To train the machine learning artificial intelligence of PERSPeCT, we used the demographic and smoking behavior characteristics of previous participants (current or former people who smoke) and their message ratings. These participants generated 16,920 ratings of 261 messages. We comparatively tested the classical algorithms k-nearest neighbors, probabilistic matrix factorization, Bayesian probabilistic matrix factorization [BPMF], collective matrix factorization, and Bayesian collective matrix factorization to identify one that provided maximal prediction accuracy (ie, we evaluated the ability of the algorithms to generalize ratings to nontraining users). We used a strong generalization protocol that involved completely separating test users from train users, learning a model using all the train users’ ratings, freezing all non–user-specific parameters, and finally training the user-specific parameters on a subset of each test user’s observed ratings [13]. To implement this protocol, users were randomly divided into 5 folds, and we then generated 3 random training and validation sets for each test fold. We further divided each test user’s ratings into 5 folds. To evaluate each method’s performance given varying levels of information about a test user, we evaluated all methods with 5, 10, and 16 of each test user’s ratings available for inference and learning of user-specific parameters. Each test user had a constant set of 4 test ratings per test fold. The validation sets were used to set the hyperparameters of each method (eg, k in k-nearest neighbors). An exhaustive grid search was used, and the hyperparameter ranges were iteratively extended to ensure that no selected hyperparameter values occurred at the end points of the search intervals. In evaluating rating prediction methods, we used a range of standard performance metrics including root mean squared error (RSME), Kendall’s tau-b, and normalized discounted cumulative gain. In all tests, BPMF was identified as the best single model in our evaluation and used in the development of PERSPeCT. For example, comparing the RSME metric between the different algorithms, there was a small but statistically significant gap (*P*=.01) between the BPMF and other algorithms as determined by a paired *t* test with Bonferroni correction. The BPMF model estimates a probability distribution over a joint embedding of users and items into complementary latent spaces. The rating a given user supplies for a given item is approximated by the expected value of the product of the latent user and item factor vectors representing the user-item pair, with the expectation taken over the uncertainty in embeddings. The result of this message selection is that the PERSPeCT recommender system outperformed a standard comparison system using simple rules to tailor messages to level of person who smokes’s motivation [13]. The proportion of days when people who smoke agreed or strongly agreed (daily rating ≥4) that the messages influenced them to quit was significantly higher in PERSPeCT (74%) than the standard comparison system (45%; *P*=.02).

### Recruitment

Recruitment of the African American PERSPeCT intervention and historical control participants was different. As such, we have conducted a detailed comparison of demographic and smoking behavior covariates. Our historical cohort of white people who smoke was recruited from the university hospital (2014) and affiliated outpatient clinics [13]. To recruit African American people who smoke in the this study (2017), we used the ResearchMatch.org online database to find eligible people who smoke by filtering our search based on ethnicity (must be African American), smoking status (must be a current person who smokes), and age (18 years or older). ResearchMatch is a free and secure online tool developed by Vanderbilt University and used by academic institutions across the country [26]. Eligible participants received a brief summary describing our study via email. Participants chose whether they would like to receive more information by clicking “I accept” on the email. ResearchMatch created a list of those who wanted additional information and research staff contacted those responders via email describing the study in more detail and offering to set up the initial baseline phone interview.

For both the African American people who smoke and comparison historical group, our inclusion criteria were the same (current people who smoke who were aged 18 years or older, English speaking, and had internet access). To confirm participation, all people who smoke had to complete an intake telephone call with study staff and complete an online registration. We provided incentives of up to $100 in Amazon gift cards for participation in the data collection.

### Experimental Procedure

As noted, all participants were required to complete an intake telephone call and log into the supportive website to complete an online consent form and a baseline questionnaire. Once registered, each participant was emailed messages selected by the PERSPeCT recommender system and asked to rate the influence of up to 30 messages within 65 days. At the end of this period, follow-up data collection was conducted with these people who smoke.

### Data Collection

During registration, people who smoke provided information on their demographic characteristics (age, sex, race, and ethnicity), smoking behaviors, prior quit attempts, and readiness to quit (I am not thinking of quitting, I am thinking of quitting, I have set a quit date, I quit today, and I have already quit) [27,28].

During the intervention, we measured ratings of messages and engagement with the supportive website. For each message, people who smoke were asked to rate message’s influence on their motivation to quit smoking. Ratings were on a 5-point Likert scale (1=strongly disagree to 5=strongly agree). Also, participants’ visits to the supportive website was tracked using online scripts.

At 30 days of follow-up, people who smoke reported the perceived impact of the 30-day PERSPeCT experience (intervention impact) and self-reported their smoking status. Intervention impact was assessed using 7 questions. These questions included actions that are known to help a person who smokes prepare to quit (talk to a doctor about quitting smoking, get support from those around you to help quit smoking, make a list of reasons to quit smoking, and use behavioral strategies like distraction or substitution) and those that could help a person who smokes actively quitting (use NRT like the patch or gum, set a quit date, and quit smoking) [29]. For the primary dependent variable, 30-day cessation, we asked, “Since starting the Quit Smoking Messaging System study have you stopped smoking for one day or longer because you were trying to quit?”

### Statistical Analyses

The analytic plan followed the flow of data collection. We first compared data collected during the active intervention and then analyzed 30-day follow-up in the African American PERSPeCT group and the comparison white PERSPeCT group.

For each day, we created a daily rating defined as the mean of the ratings provided by all people who smoke in that group that day. We graphed this data by day and reported the percentage of days where the African Americans rated the message higher than whites. We then compared mean of daily ratings between African American and white people who smoke using a *t* test. We measured engagement with the supportive website by comparing mean number of visits to the Web-assisted tobacco intervention between African American and white people who smoke using the *t* test statistic. We dichotomized (agree or strongly agree versus other) the responses to each of the seven questions that assessed intervention impact, comparing African American and white PERSPeCT groups using the chi-square statistic. We calculated the percentage of participants who reported 30-day cessation. In the logistic regression analyses, 30-day cessation (ie, quit for at least 1 day) was considered the dependent variable (yes or no). Participant’s race was the independent variable, and we adjusted for covariates that were significantly different between the two groups. Finally, we conducted a formal mediation analysis to evaluate whether difference in ratings during the intervention (comparing African Americans and whites) mediated the difference in 30-day smoking cessation.

For both the during intervention and 30-day follow-up analyses, we used Stata statistical software (StataCorp LLC). For the mediation analysis, we used the Stata medeff command and sought to quantify the effect of PERSPeCT that operates through the path of differential experience with messages (as measured by daily ratings). For the mediation analysis, we first fit a linear regression model evaluating the association of ethnicity and the mediator (daily ratings) and then a second logistic regression model with the main outcome as 30-day smoking cessation. The independent variable for the main model was ethnicity, and the mediator was daily ratings. We report the percentage of the total effect mediated by daily ratings.

## Results

### Patient Characteristics

African American people who smoke were significantly more likely to allow smoking in the home compared with whites (*P*=.002); all other characteristics were balanced between the two groups (see Table 1).

**Table 1:** Demographic characteristics and smoking behavior at baseline.

Characteristic		White people who smoke (n=64), n (%)	African American people who smoke (n=55), n (%)	*P* value
**Gender**				**.80**
	Male	24 (37)	22 (40)	
	Female	40 (63)	33 (60)	
**Age in years**				**.76^a^**
	19-34	21 (33)	17 (31)	
	35-44	17(27)	14 (25)	
	45+	26(40)	24 (44)	
**Education**				**.84**
	Other^b^	51 (80)	43 (78)	
	Advanced college degree	13 (20)	47 (22)	
**Ethnicity**				**.23**
	Not Hispanic	59 (97)	52 (95)	
	Hispanic or Latino	2 (3)	3 (5)	
**Allow smoking at home**				**.002**
	No	40 (63)	19 (35)	
	Yes	24 (37)	36 (65)	
**Ever visited a smoking cessation website**				**.37**
	No	52(81)	48 (87)	
	Yes	12 (19)	7 (13)	
**During the past 12 months, stopped smoking for one day or longer because you were trying to quit smoking**				**.85**
	No	36 (56)	30 (55)	
	Yes	28 (44)	25 (45)	
**Current smoking status**				**.87**
	Not actively quitting	52 (81)	44 (80)	
	Actively quitting	12 (19)	11 (20)	

^a^Tested for trend using the Mantel-Haenszel method (mHodds command in Stata).

^b^Some high school, high school diploma, some college or technical school.

### During Intervention

**Figure 1:**
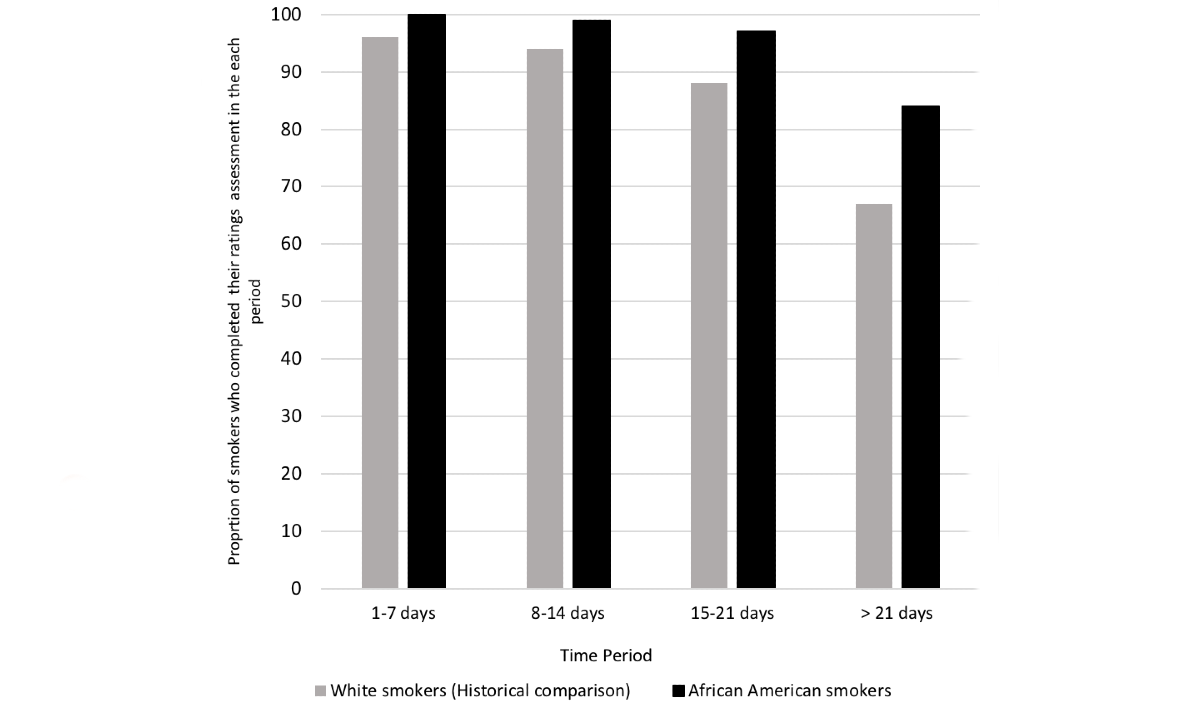
Proportion of people who smoke completing ratings assessments by study time periods.

**Figure 2:**
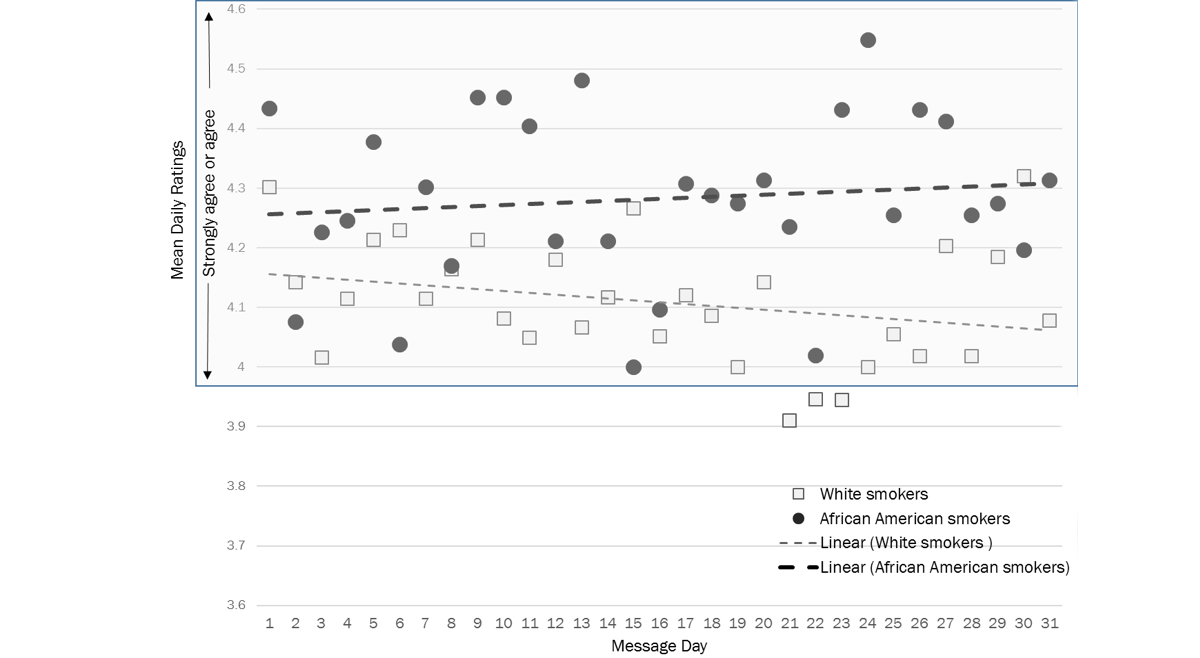
African American and white people who smoke mean message ratings over time.

More African American people who smoke rated all 30 messages than white people who smoke (Figure 1). Using daily message rating averages, African American people who smoke rated messages (daily message rating ≥4) on all days (100% of the 30 days of messages) compared with 77% (23/30) of days for the white people who smoke (*P*<.001; Figure 2). Daily mean ratings were higher for African American than white people who smoke on 26 of the 30 days (87% [26/30] African Americans higher vs 13% [4/30] of days where whites had higher daily rating; *P*<.001). Overall, daily message ratings were higher for African American people who smoke (mean 4.27 [SD .02]; range 4.23-4.31) than white people who smoke (mean 4.10 [SD .11]; range 4.05-4.15; *P*<.001).

African American people who smoke had significantly more visits to the Web-assisted tobacco intervention compared with white people who smoke (African American mean 5.5 [SD 1.3]; range 3.0-8.0 vs white mean 1.5 [SD 0.1]; range 1.2-1.7; *P*<.001).

### Follow-Up

**Figure 3:**
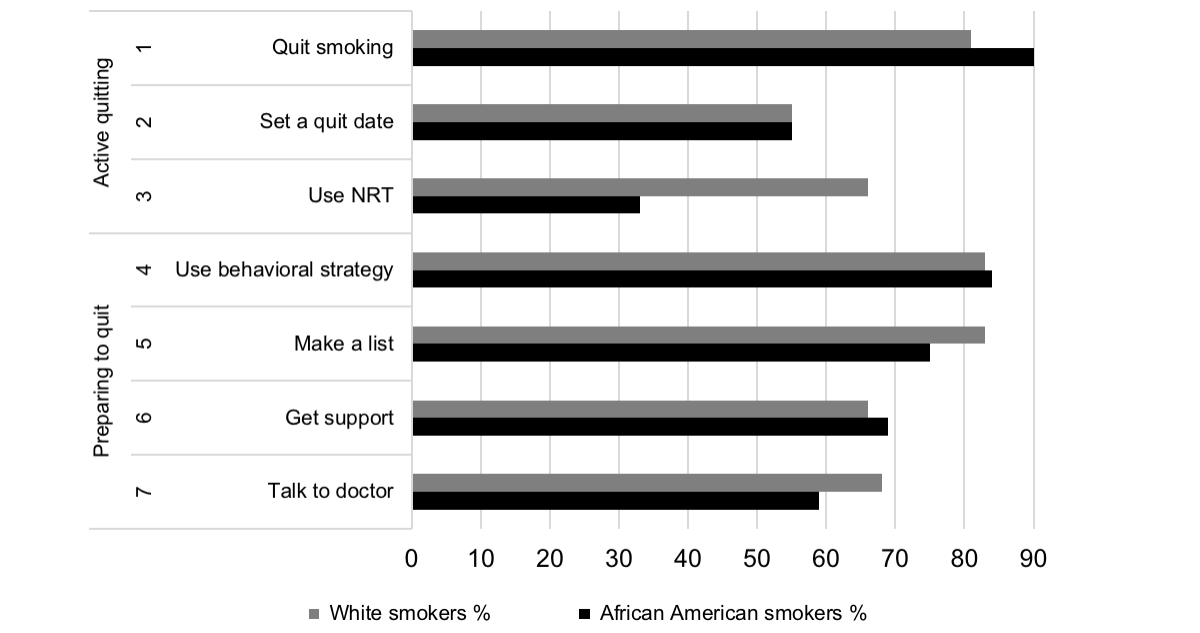
Perceived influence on quitting strategies between African-American and white people who smoke.

Perceived influence of use of NRT was significantly lower among the African American people who smoke compared with the white poeple who smoke (66% [35/53] vs 33% [17/51], *P*=.001; Figure 3). More African American people who smoke reported a perceived influence to quit smoking than white people who smoke, but this was not significant (90% [46/51] vs 81% [43/53], *P*=.19).

African American people who smoke were significantly more likely to quit than white people who smoke (African American 59% [30/51], white 38% [19/50], *P*=.03). In the unadjusted logistic model, compared with white people who smoke, odds of quitting were significantly higher for African Americans (odds ratio [OR] 2.3; 95% CI 1.04-5.53). The result did not change after adjusting for allowing smoking at home (OR 2.4; 95% CI 1.04-5.53). In the secondary mediation analysis, we again found that daily ratings were higher among African Americans versus whites (linear regression beta=0.49, *P*=.002), and experience with the PERSPeCT messages (measured by daily ratings) mediated 42% of the total effect of ethnicity on 30-day smoking cessation.

## Discussion

### Principal Findings

Consistently across the 30 days, message ratings of African American people who smoke were higher than those of white people who smoke. Further, mean African American people who smoke ratings started higher than white people who smoke, and ratings within this group increased over time. On the contrary, mean white people who smoke ratings started lower than African American people who smoke, and ratings within this group decreased over time. Self-reported perceived influence of the intervention on use of NRT was lower among African American people who smoke than white people who smoke. Intervention engagement was higher among African Americans people who smoke compared with white people who smoke. African American people who smoke were significantly more likely to report 30-day cessation as compared with white people who smoke, and this different was mediated by experience with PERSPeCT.

There may be several reasons for the higher ratings of African American people who smoke than white people who smoek in the study. The ability to influence the message a perosn who smokes receives (by rating the message) may have provided an enhanced feeling of control over the intervention. African American people who smoke may have been more attracted to this increased autonomy, the extent to which a behavior or course of action is personally endorsed and engaged [30], than white people who smoke. A potential advantage of using a recommender system is that the machine learning algorithms can learn from user feedback and improve the selection of messages over time [9]. In our study, only the ratings of African American people who smoke increased over time, whereas for white people who smoke the ratings decreased over time. There may have been a symbiotic relationship between intervention engagement and message selection. More African American people who smoke rated all messages than white people who smoek. The increased feedback may have resulted in better message selection, which then resulted in higher ratings and vice versa. African Americans may have benefited from increased engagement with the Web-assisted tobacco intervention, which made them more receptive to the messages from the system. Previous findings have shown that increasing the personalization of a message increases both the relevance and relatedness of the message to the user [1]. The increased engagement with the supportive website may also reflect higher motivation among the African American people who smoke than the white people who smoke resulting in higher ratings.

The lower perceived intervention impact for use of NRT among African American compared with white people who smoke may have highlighted a potential unintended consequence of using recommender systems. Evidence shows African American people who smoke are less likely to successfully quit than white people who smoke [15]. A primary reason for this is the low use of NRT by African American people who smoke [31]. Several reasons have been identified for the low use of NRT by African American people who smoke, including misinformation about the safety and addictive potential of NRT [32], as well as possible interactions with other medications. If the system targeting African American people who smoke is based primarily on user feedback (as PERSPeCT is), such a system may never select messages that address the use of NRT if the user provides low rating for these messages. This calls for the use of hybrid systems that combine a rule-based and recommender approach for CTHC, incorporating both their strengths. Optimal strategies for developing this hybrid approach (eg, when the message selection should be rule-based versus a recommender approach) needs to be tested.

The 30-day cessation data highlights PERSPeCT’s potential as a cessation intervention for African American people who smoke. Culturally tailored materials have been shown to enhance the effectiveness of previous trials [33]. Although we did not alter messages to the target population, we hoped that the use of individual feedback from African American users may have improved the system’s ability to select messages that reflect values and practices specific to other African Americans people who smoke. As noted above, African American people who smoke were more engaged with the ratings assessment, which may have resulted in better message selection than in white people who smoke. The difference in cessation results was despite African American people who smokes’ lower perceived influence for use of NRT, a known facilitator of cessation. Also, allowing smoking in the home, a known barrier to smoking cessation, was more prevalent in the African American sample, and this makes the success of PERSPeCT even more promising. Further studies are needed to test the long-term effect of the recommender system to promote cessation.

### Limitations

This study has some limitations. The difference in recruitment approaches may have influenced the results of our study. As noted, we were able to recruit white people who smoke within the local area (Central Massachusetts and surrounding areas). To recruit African Americans, we had to recruit in multiple states using ResearchMatch. However, as Table 1 noted, allowing smoking at home was the only difference between the two study groups. In the 30-day cessation analysis, we adjusted for allowing smoking at home, and the results were the same as the unadjusted model. Second, the sample size may have been insufficient to detect cessation differences that may truly exist between white and African American people who smoke. Study results should therefore be interpreted with caution as a larger study is needed to confirm our findings. Third, as appropriate for a pilot, the study only assessed 30-day cessation and did not follow the people who smoke over 6 months or 1 year. The longer term is considered an appropriate time window to assess the impact of intervention on smoking cessation [34]. Fourth, the lack of random assignment creates uncertainties in inferring that the PERSPeCT recommender system resulted in the observed differences between white and African American people who smoke. As noted previously, we used a historical comparison (white people who smoke) to compare to the participants (African American people who smoke) enrolled in this study. Systematic differences in characteristics between the two study groups may have implications for selection bias. Future studies that can effectively account for dissimilarities between the two groups are needed to make causal inferences [35]. Fifth, use of a historical cohort of white people who smoke as a comparison group poses a temporal challenge; people who smoke recruited in 2014 may not accurately represent people who smokes’ perspectives in 2017 or current perspectives [36]. Therefore, observed group differences among people who smoke may be a result of time-based differences (>2 years) rather than true group differences. Despite these limitations, the results of our exploratory study indicate potential for testing of the recommender system with African American people who smoke in a larger study.

### Conclusions

Few systems have been able to select messages of higher influence, increasing engagement with the system and self-reported 30-day cessation rates. Additional innovations such as using a hybrid rule and recommender approach may be needed to effectively engage African American people who smoke while also motivating the use of NRT and other effective treatments. Also, since recommender systems can learn from user feedback and adapt over time, the system might be even more effective over a longer duration (6 months or a year). Future research is needed to test the long-term effectiveness of using a recommender system CTHC approach for smoking cessation in African American people who smoke to assess the true impact of PERSPeCT.

## Abbreviations

BPMF: Bayesian probabilistic matrix factorization
CTHC: computer-tailored health communication
NRT: nicotine replacement therapy
OR: odds ratio
PERSPeCT: Patient Experience Recommender System for Persuasive Communication Tailoring
RMSE: root mean square error
